# Tissue Transglutaminase-Regulated Transformed Growth Factor-*β*1 in the Parasite Links *Schistosoma japonicum* Infection with Liver Fibrosis

**DOI:** 10.1155/2015/659378

**Published:** 2015-06-23

**Authors:** Juanjuan Tang, Xunmin Zhu, Jingjing Zhao, Mingchiu Fung, Yinyan Li, Zhiyan Gao, Suikai Yan, Xiaomin Li, Xiaofang Ji, Fang Su, Zi Li

**Affiliations:** ^1^Guangzhou Hoffmann Institute of Immunology, School of Basic Sciences, Guangzhou Medical University, Guangzhou 510182, China; ^2^Morphology Department, School of Basic Sciences, Guangzhou Medical University, Guangzhou 510182, China; ^3^The First Affiliated Hospital of Guangzhou Medical University, Guangzhou 510120, China; ^4^School of Life Sciences, The Chinese University of Hong Kong, Hong Kong

## Abstract

Transforming growth factor (TGF-*β*1) is among the strongest factors of liver fibrogenesis, but its association with *Schistosoma*-caused liver fibrosis is controversial. Tissue transglutaminase (tTG) is the principal enzyme controlling TGF-*β*1 maturation and contributes to *Sj*-infected liver fibrosis. Here we aim to explore the consistency between tTG and TGF-*β*1 and TGF-*β*1 source and its correlation with liver fibrosis after *Sj*-infection. TGF-*β*1 was upregulated at weeks 6 and 8 upon liver fibrosis induction. During tTG inhibition, TGF-*β*1 level decreased in sera and liver of infected mice. TGF-*β*1 showed positive staining in liver containing *Sj* adult worms and eggs. TGF-*β*1 was also detected in *Sj* adult worm sections, soluble egg antigen and *Sj* adult worm antigen, and adult worms' culture medium. The TGF-*β*1 mature peptide cDNA sequence and its extended sequence were amplified through RT-PCR and RACE-PCR using adult worms as template, and sequence is analyzed and loaded to NCBI GenBank (number GQ338152.1). TGF-*β*1 transcript in *Sj* eggs was higher than in adult worms. In *Sj*-infected liver, transcriptional level of TGF-*β*1 from *Sj*, but not mouse liver, correlated with liver fibrosis extent. This study provides evidence that tTG regulates TGF-*β*1 and illustrates the importance of targeting tTG in treating *Sj* infection-induced fibrosis.

## 1. Introduction

Schistosomiasis is one of the nine neglected tropical diseases that received much attention over the last several years. After* Schistosoma* cercariae penetrate the hosts' skin and develop into adult worms, they reside in tributaries of the portal vasculature where they continuously release eggs. The portal blood flow then carries the eggs into the liver where they induce production of inflammatory granuloma and, subsequently, tissue repair and fibrosis.* Schistosoma japonicum* (*Sj*) mainly damages mammalian hosts by producing liver granuloma and fibrosis [1].

Transforming growth factor (TGF-*β*1) is one of the strongest factors that lead to liver fibrosis. TGF-*β*1 promotes hepatic stellate cell (HSC) proliferation and collagen synthesis in the activated HSC [2–4] or modulates deposition of extracellular matrix (ECM) components and immune functions [5]. However, the relationship between TGF-*β*1, liver fibrosis, and* Schistosoma* infection is controversial. Alves Oliveira et al. [6] and Kaviratne et al. [7] have demonstrated that IL-13, but not TGF-*β*1, is strongly associated with fibrosis during* S. mansoni* (*Sm*) infection. However, Techau et al. [8] discovered that pigs prenatally exposed to* Sj* showed higher levels of TGF-*β*1 mRNA expression in the liver than postnatally infected and noninfected pigs. TGF-*β*1 has sometimes been accepted as the key factor inducing liver granuloma and fibrosis during* Sj* infection because some researchers have recognized TGF-*β*1 inhibition as one of the factors that can be used to evaluate the antifibrotic effects of drugs on hosts infected with* Sj* [9–11]. However, systemic studies that reveal whether liver fibrosis caused by* Sj* infection is dependent or not dependent on TGF-*β*1 are lacking.

In vertebrates, the TGF-*β* superfamily is a structurally conserved but functionally diverse group of proteins with at least 35 members, including the prototypic TGF-*β* subfamily (comprising TGF-*β*1, TGF-*β*2, and TGF-*β*3), an extensive bone morphogenetic protein (BMP) subfamily (with 20 members), the growth and differentiation factor subfamily (at least 9 members), and the activin/inhibin subfamily (InACT). A common feature shared by the members of this family is that the mature bioactive forms are homo- or heterodimers corresponding to the cleaved carboxyterminal regions of larger preproproteins [12]. The activated TGF-*β*1 binding with specific receptors in the cell membrane through the Smad signal transduction pathways plays the biological role [13]. Some members of TGF-*β* superfamily, including InACT, BMP, receptors of TGF-*β* [14–18] and Smad1, Smad2, and Smad4, and their signaling pathway-associated molecules have been identified in* Schistosoma* [19–22]. InAct plays important roles in* Sm* development and embryogenesis [23]. In addition, Hirata et al. [24] revealed the expression of TGF-*β*-like molecules in* Sj* cercariae, schistosomula, eggs, and adult worms by using antibodies against anti-mouse TGF-*β*1, TGF-*β*2, and TGF-*β*3, respectively. However no study has revealed that members of TGF-*β* subfamily exist in* Sj*, as well as their roles in the parasite development or pathogenesis, even though genomes of* Sj* and* Sm* have already been analyzed [25, 26].

Molecular mechanisms of host-parasite interaction are complex and involve much molecular cross talk, including ligands and receptors, substrates, and enzymes, which are either from the host or from the parasite. Up to now, many studies have indicated that tissue transglutaminase (tTG) and TGF-*β*1 are closely related. TGF-*β*1 dimer is synthesized intracellularly and combines with latency-associated peptide (LAP) to form the small latent TGF-*β* complex (SLC). The mature inactive SLC then forms the large latent TGF-*β* complex (LLC) by covalent bonding with the large latent TGF-*β* binding protein (LTBP-1) and is stored in the ECM [27, 28]. Latent TGF-*β* activation in the ECM involves tTG as the principal enzyme that covalently cross-links LBTP to major ECM proteins, such as fibronectin, thereby controlling the rate of TGF-*β* maturation [29–31]. Upregulation of extracellular tTG increases the levels of active TGF-*β* both in cell-culture models and in vivo in various pathological states [32, 33].

Our previous research showed that tTG is involved in the development of* Sj-*infection-induced liver fibrosis in mice, and the underlying mechanism may be associated with tTG-regulated IL-13 expression [34]. In this study, we investigated the association between tTG and TGF-*β*1 that originated from the host or from* Sj* using* Sj*-infected mice as liver fibrosis model. We showed that tTG-regulated TGF-*β*1 in the parasite is related to mouse liver fibrosis after* Sj*-infection.

## 2. Materials and Methods

### 2.1. Ethics Statement

This study was performed in strict accordance with the recommendations of the Guide for the Care and Use of Laboratory Animals of State Scientific and Technological Commission. The protocol was approved by the Committee on the Ethics of Animal Experiments of the University of Guangzhou Medical University (permit number: SCXK(Guangdong)2011-0029). All surgeries were performed under sodium pentobarbital anesthesia, and every effort was made to minimize suffering.

### 2.2. Parasites, Animals, and Culture Medium of* Sj* Adult Worms

Female BABL/c mice (6 weeks old to 8 weeks old; from the Experimental Animal Center of Sun Yat-Sen University, Guangzhou, China) were maintained according to the guidelines approved by the Guangzhou Medical University Animal Experiment and Care Committee. Cercariae of* Sj* Chinese mainland strain were obtained from the infected* Oncomelania hupensis* (Jiangsu Institute for Schistosomiasis Control, Wuxi, China). Adult schistosomes were recovered by hepatic-portal perfusion from BABL/c mice that had been percutaneously exposed to 20 ± 3 cercariae. Adult parasites and eggs were collected and were maintained in phosphate-buffered saline (PBS) for soluble worm antigen (SWA) and soluble egg antigen (SEA) preparation. Twenty pairs of freshly washed adult worms were transferred to 2 mL RPMI 1640 medium supplemented with 1 mM glutamine, 1000 units/mL penicillin, and 1000 *μ*g/mL streptomycin for 2 h. Worms were finally cultured in 2 mL sterile RPMI 1640 medium supplemented with 20% sterile fetal bovine serum (FBS), 1 mM glutamine, 100 units/mL penicillin, and 100 *μ*g/mL streptomycin for 16 h. The adult* Sj* culture medium and negative control medium (sterile RPMI 1640 medium supplemented with 20% FBS, 1 mM glutamine, 100 units/mL penicillin, and 100 *μ*g/mL streptomycin) were collected.

### 2.3. Reagents

TGF-*β*1 ELISA kit was obtained from R&D systems. The following antibodies were used for Western blot analysis or immunohistochemistry (IHC) assay: anti-TGF-*β*1(V) (sc-146, Santa Cruz Biotechnology), anti-GAPDH (Cell Signaling Technology), anti-alpha SMA (BOSTER), anti-Smad2 (sc-101153, Santa Cruz Biotechnology), and anti-phosphospecific Smad2 (sre465/476, MILLIPORE).

### 2.4. Parasite Infection, Cystamine (CTM) Administration, and Sample Collection

Forty BABL/c mice were infected cutaneously with 20 ± 3* Sj* cercariae for 5, 6, 8, or 12 weeks (10 mice in each time course), and 10 uninfected mice served as the control. CTM (Sigma-Aldrich, St. Louis, USA, tTG inhibitor) treatment in mice was shown in our previous study [34]. CTM (10^−2 ^mM) was administered in each mouse once per day for 7 d, whereas PBS was used as control. Blood sera for ELISA were collected from each group by cutting the caudal vein of the mice. Perfusions of the hepatic portal system of* Sj*-infected mice were performed to collect adult worms, as described previously. Meanwhile, liver lobes were prepared for Western blot analysis, IHC or RT-PCR, and qPCR. Mouse infection, CTM administration, and sample collection were repeated at least twice.

### 2.5. TGF-*β*1 Detection by ELISA

Blood samples were collected by cutting the tail veins of mice in each group and placed into EP tube with 1000 IU/mL heparin (10 *μ*L), after which blood plasma was collected (3000 rpm, 10 min centrifugation). Blood plasma was diluted 1 : 4 ratio and was immediately used in experiments. TGF-*β*1 level were detected using the mouse DuoSet ELISA Development kit (R&D Systems: DY1679) according to the manufacturer's instructions.

### 2.6. Western Blot

The liver of* Sj-*infected mouse was collected and was ground into powder in liquid nitrogen, and moderate amount of protein lysis solution (RIPA from Shanghai Bocai Biological Technology Co., Ltd.) was added for liver tissue protein preparation. Protein concentration was determined by Bradford assay (Bio-Rad, Redmond, WA). Tissue lysates (30 *μ*g) were separated by 10% SDS-PAGE and then transferred onto polyvinylidene fluoride membranes (Amersham, Bucks, UK). The membranes were blocked with 5% nonfat dried milk before incubation with target-specific antibodies. Protein bands were detected with ECL reagents.

### 2.7. RT-PCR, Rapid Amplification of cDNA Ends-PCR (RACE-PCR), and Real-Time Quantitative Polymerase Chain Reaction (Q-PCR)

Liver tissues of mice or* Sj* adult worms from* Sj*-infected mice were homogenized in Trizol (Invitrogen, Carlsbad, CA), and total RNA was extracted according to the manufacturer's protocol. RNA purity was assessed by spectrophotometry. Reverse transcription reactions for cDNA synthesis were performed using PrimeScript RT Master Mix (TAKARA). Relative expression level of mRNA was determined by Q-PCR with SYBR Green I PCR Master (TAKARA) using ABI7500. Data were normalized with mouse GAPDH and* Sj* tubulin-*α*. PCR products were analyzed by electrophoresis on 1% agarose gels containing ethidium bromide, and Q-PCR results were expressed as fold amplification using the 2^−ΔΔCt^ method. Each experiment was repeated three times.

### 2.8. Statistics

All experiments were repeated at least twice with similar results. Data were compared by Student's *t*-test. Results were expressed as mean ± SD. *P* < 0.05 was considered significant.

## 3. Results

### 3.1. TGF-*β*1 Is Upregulated in* Sj*-Infected Mice

**Figure 1:**
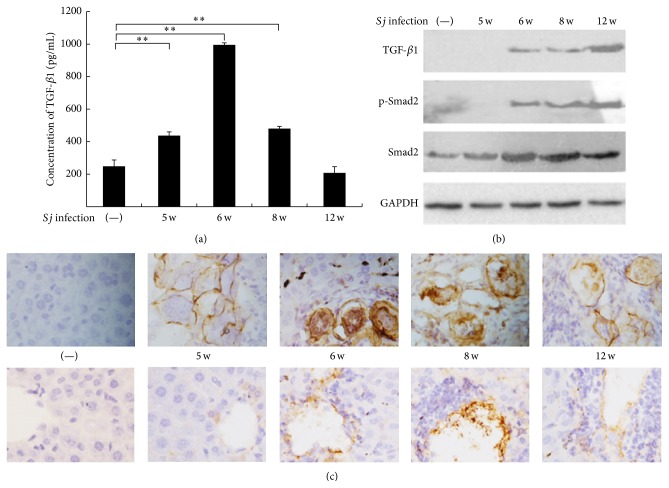
TGF-*β*1 level increased gradually in mice liver during the courses of* Sj* infection. (a) TGF-*β*1 level in serum was determined by ELISA in BALB/c mice with 20 ± 3 infective* Sj* cercariae for 5, 6, 8, and 12 weeks, and uninfected mice were used as control. Data are shown as means ± SD of 10 mice/group. Experiment was performed four times (^*∗*^ *P* < 0.05; and ^*∗∗*^ *P* < 0.01 compared with uninfected group). (b) Equal amounts of proteins of mouse liver tissue lysates at indicated time points were used in the Western blot assay to detect protein expression levels of TGF-*β*1, p-Smad2, and Smad2. GAPDH was used as loading control. (c) Mouse livers at indicated time points were fixed in paraformaldehyde, embedded in paraffin, and then sliced and immunohistochemically stained for TGF-*β*1. Representative staining for TGF-*β*1 is shown at ×400 magnification. Top: TGF-*β*1 expression in egg,* Sj* egg granuloma, and the surrounding tissue. Bottom: TGF-*β*1 expression in hepatic cell and liver tissue around the liver sinusoid.

**Figure 2:**
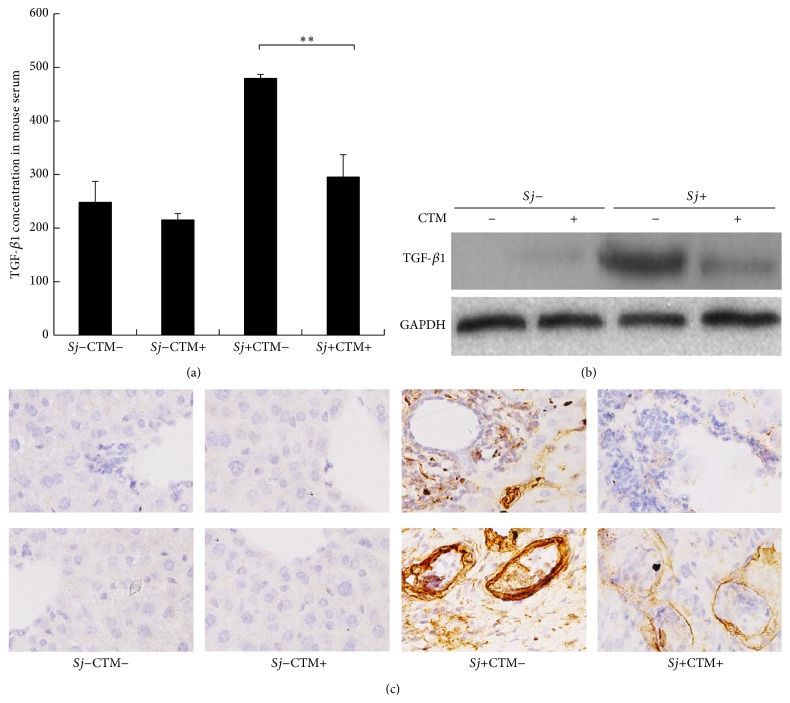
CTM reduced TGF-*β*1 expression profile in the liver of* Sj*-infected mice. tTG activity of BABL/c mouse liver was inhibited by CTM intraperitoneal injection from day 3 to day 10 after* Sj* infection. Mice were sacrificed at week 8 after infection. (a) Activated TGF-*β*1 concentration in mouse serum of normal or* Sj-*infected mice with or without CTM treatment was detected using ELISA. Data were presented as mean ± SD from 10 mice per each group. ^*∗*^ *P* < 0.05; and ^*∗∗*^ *P* < 0.01. (b) Activated TGF-*β*1 protein levels in mouse liver of normal or* Sj*-infected mice with or without CTM treatment were detected by Western blot analysis, and GAPDH was used as the internal control. (c) Mouse liver samples collected at indicated time points were fixed in paraformaldehyde, embedded in paraffin, sliced, and immunohistochemically stained for TGF-*β*1. Representative stainings for TGF-*β*1 were shown at 40x magnification. Bottom: TGF-*β*1 expression in egg,* Sj* egg granuloma, and the surrounding tissue. Top: TGF-*β*1 expression in hepatic cell and liver tissue around the liver sinusoid; “−” = without, “+” = with.

We previously reported a high extent of post-*Sj-*infection hepatic fibrosis in mice. Liver granuloma began at week 5, and fibrosis progressed most seriously at week 8, whereas chronic liver fibrosis appeared at week 12 [34]. TGF-*β*1 was usually the key factor in inducing liver fibrosis compared with other causes [2, 3]. TGF-*β*1 concentration in mouse serum increased 5, 6, and 8 weeks after* Sj* infection, and the highest level was observed at week 6 (Figure 1(a)). Western blot analysis and IHC assay revealed that TGF-*β*1 protein level in* Sj*-infected mouse liver also increased (Figures 1(b) and 2(c)). In addition, Smad2, the downstream signaling protein of TGF-*β*1 pathway, was also activated in mice liver after* Sj* infection (Figure 1(b)). TGF-*β*1 could be involved in liver fibrosis in a Smad2-dependent manner during* Sj* infection. TGF-*β*1 was localized either in the cells of blood vessels where* Sj* adult worms reside or in the eggs of* Sj* and in the cells of liver tissue where eggs are deposited (Figure 1(c)). The results suggested that TGF-*β*1 likely promoted hepatic fibrosis after* Sj* infection.

### 3.2. Mature TGF-*β*1 Level Is Decreased along with Alleviation of tTG Activity

To clarify whether tTG induces TGF-*β*1 maturation during* Sj* infection, tTG activity inhibitor CTM was used to block tTG activity. The extent of liver fibrosis was suppressed after* Sj*-infected mice were treated with CTM, and no effect was observed in untreated mice [34]. ELISA results showed that the concentrations of TGF-*β*1 mature peptide were 296.21 and 480.35 pg/mL in mouse sera with and without CTM treatment, respectively (Figure 2(a)) (*P* < 0.05). Western blot analysis results revealed that TGF-*β*1 protein expression level in CTM-treated* Sj*-infected mice liver in situ was lower compared with that in* Sj*-infected mice that were not subjected to CTM treatment (Figure 2(b)). IHC assay results showed that, in mice liver in situ, the intensity of positive stain, which indicated active TGF-*β*1, was remarkably reduced in CTM-treated* Sj*-infected mice liver compared with* Sj*-infected mice without CTM treatment (Figure 2(c)). Moreover, this reduction was mainly observed around hepatic sinusoids where* Sj* adult worms reside, as well as around and in egg granulomas where* Sj* eggs are deposited. TGF-*β*1 in* Sj* eggs was also reduced in* Sj*-infected mice subjected to CTM treatment (Figure 2(c)). These results indicated that tTG-regulated TGF-*β*1 promoted hepatic fibrosis in mice during* Sj* infection, and TGF-*β*1 proteins located in* Sj* are partially regulated by tTG of host origin.

### 3.3. TGF-*β*1 Protein Was Detected in* Sj*

**Figure 3:**
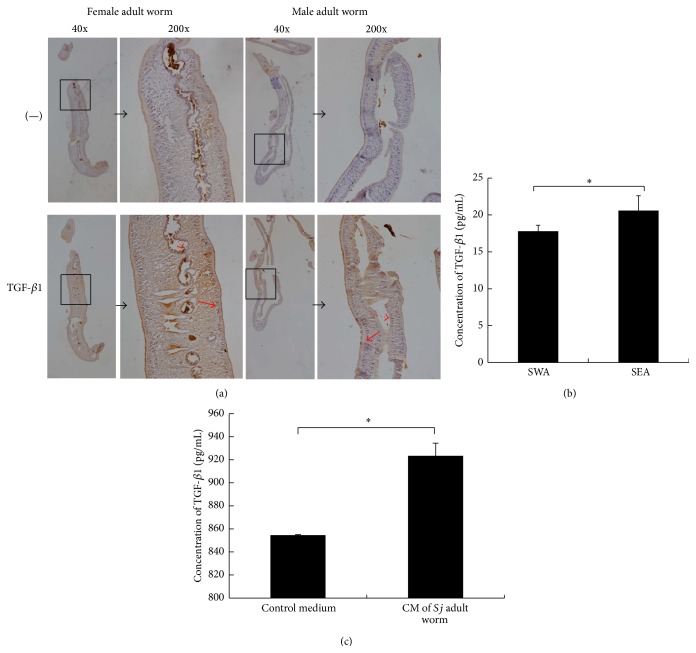
TGF-*β*1 protein was expressed in* Sj* and was secreted. (a)* Sj* adult female and male worms were fixed in paraformaldehyde, embedded in paraffin, and then sliced and stained for TGF-*β*1. Representative staining (brown) is shown at 40x magnification (large panel) and ×200 (inset). Dotted red arrow: gut epithelial cells; solid red arrow: subtegumental cells. (b) Equal amounts of* Sj* soluble adult worm antigen and soluble egg antigen were tested for TGF-*β*1 by ELISA. Data were shown as means ± SD. Experiment was performed four times (^*∗*^ *P* < 0.05; and ^*∗∗*^ *P* < 0.01 compared with negative PBS control). (c) Twenty pairs of adult worms were freshly collected, washed thrice using PBS, transferred into 2 mL sterile RPMI 1640 medium supplemented with 1 mM glutamine, 1000 units/mL penicillin, and 1000 *μ*g/mL streptomycin for 2 h, and finally cultured in 2 mL sterile RPMI 1640 medium supplemented with 20% FBS, 1 mM glutamine, 100 units/mL penicillin, and 100 *μ*g/mL streptomycin for 16 h.* Sj* worm culture medium was collected for TGF-*β*1 detection by ELISA, and condition medium (sterile RPMI 1640 medium supplemented with 20% FBS, 1 mM glutamine, 100 units/mL penicillin, and 100 *μ*g/mL streptomycin) was used as control. Experiment was performed four times (^*∗*^ *P* < 0.05; and ^*∗∗*^ *P* < 0.01 compared with control medium without* Sj* adult worms).

Similar to the findings of Hirata et al. [24], our results showed that TGF-*β* subfamily immunoreactive molecules are probably expressed in adult worms and eggs of* Sj* (Figure 2(c)). We validated these results through an IHC assay using sections of male and female adult worms (Figure 3(a)). Moreover, we detected TGF-*β*1 protein in the SEA, SWA of* Sj*, and in the culture medium of* Sj* adult worm using ELISA (Figures 3(b) and 3(c)). Figure 3(a) shows that TGF-*β*1 immunoreactivity was apparent in subtegumental cells and the lining gut epithelial cells of male and female worms, especially in female worms. TGF-*β*1 concentrations in SEA and SWA were 17.9 and 20.7 pg/mL, respectively (Figure 3(b)). Furthermore, higher concentration of TGF-*β*1 was secreted in culture medium of adult worms than in the control medium (Figure 3(c)).

### 3.4. Amplification of TGF-*β*1 cDNA Sequence Selectively Using* Sj* as Template

**Figure 4:**
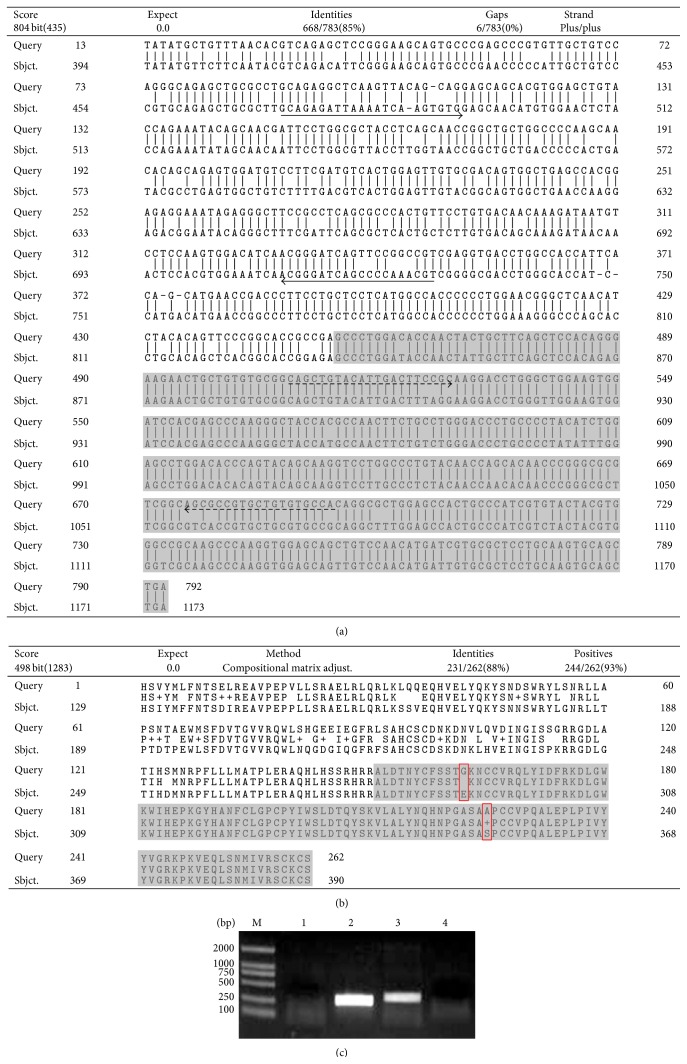
TGF-*β*1 gene in* Sj* was cloned and identified. (a) TGF-*β*1 mature peptide cDNA in* Sj* was amplified using* Sj* adult worm cDNA collected from infected mice as template and primers designed according to mouse TGF-*β*1 mature peptide sequence, and the 5′ and 3′ ends of TGF-*β*1 in* Sj* were extended via RACE. The alignment of TGF-*β*1 cDNA sequences of* Sj* and mouse is shown (Query is* Sj* TGF-*β*1 and Sbjct. is mouse TGF-*β*1 cDNA sequence). Solid lines: specific mouse TGF-*β*1 primer pairs for PCR amplification; dotted lines: specific* Sj* TGF-*β*1 primer pairs for PCR amplification. Gray: TGF-*β*1 mature peptide cDNA sequence. (b) The alignment of TGF-*β*1 amino acid sequences of* Sj* and mouse is shown (Query is* Sj* TGF-*β*1 and Sbjct. is mouse TGF-*β*1 amino acid sequence). Gray: TGF-*β*1 mature peptide amino acid sequence. (c) TGF-*β*1 primers of mouse- or* Sj*-specific source were identified through PCR using* Sj* adult worms or normal mice liver cDNA as template, respectively. M: DL2000 DNA Marker; 1: mouse cDNA as template and specific* Sj* TGF-*β*1 primer; 2: mouse cDNA as template and specific mouse TGF-*β*1 primer; 3:* Sj* cDNA as template and specific* Sj* TGF-*β*1 primer; 4:* Sj* cDNA as template and specific mouse TGF-*β*1 primer.

To clarify whether or not TGF-*β*1 gene exists in* Sj*, we designed a pair of primers according to the cDNA sequence of mouse TGF-*β*1 mature peptide because the amino acid sequence of TGF-*β*1 mature peptide is highly conserved. This primer pair and* Sj* adult worm cDNA isolated from mice as template were used for PCR amplification of* Sj* TGF-*β*1 mature peptide cDNA, and a 339 bp product was obtained, sequenced, and analyzed using bioinformatics. The 5′ and 3′ ends of the fragment were extended via RACE-PCR using primers designed within the known sequence. The extended TGF-*β*1 cDNA sequence (792 bp long) was loaded into National Center for Biotechnology Information (NCBI) GenBank with number GQ338152.1. The deduced amino acid sequence comprised 263 amino acid residues and contained partial sequence of TGF-*β*1 propeptide and complete TGF-*β*1-like domain. The nucleotide sequence of extended TGF-*β*1 gene from* Sj* was 85% identical to that from mouse (Figure 4(a)), whereas the amino acid sequence of TGF-*β*1 was 88% identical (Figure 4(b)), as revealed by BLAST search results. The nucleotide sequence of* Sj* TGF-*β*1 mature peptide was different from that of mouse by multiple nucleotides, but the amino acid sequence of* Sj* TGF-*β*1 mature peptide had merely two amino acid differences compared with that of mouse (Figure 4(b)). To identify species-specific TGF-*β*1, we designed and identified primers of* Sj* and mouse-specific primers (the primer sequences have been underlined in solid and dashed lines, resp., as shown in Figure 4(a)).  Figure 4(c) shows that positive bands appeared in the agarose gel only when we used specific primer pairs and the corresponding templates for PCR amplification. Moreover, the transcription level of TGF-*β*1 was higher in eggs than in adult* Sj* worms. These results suggested the existence of TGF-*β*1 gene in* Sj* adult worms and eggs.

### 3.5. High TGF-*β*1 Transcription Level in* Sj* Was Consistent with the Extent of Liver Fibrosis in Mice

**Figure 5:**
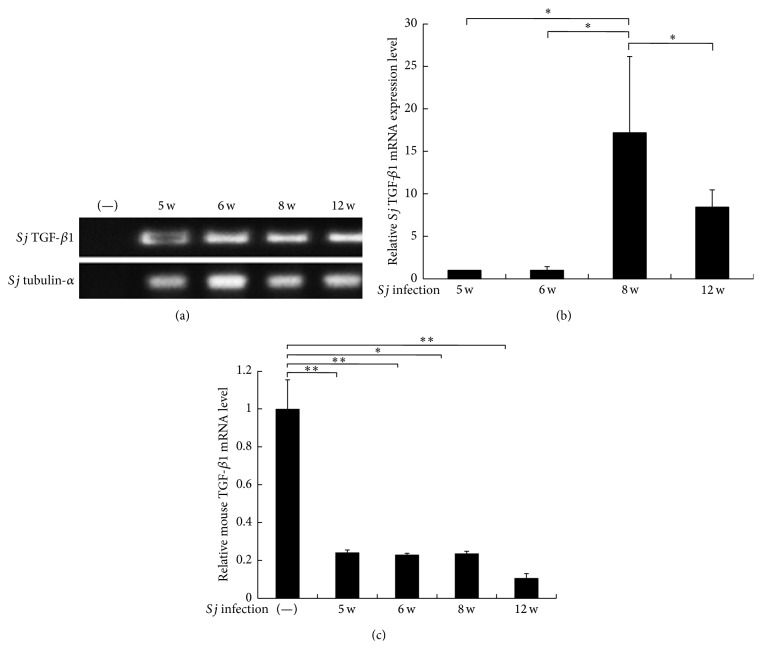
TGF-*β*1 is transcribed in* Sj* and* Sj*-infected mouse liver. Equal amounts of total RNA from left lobes of BALB/c mice liver with 20 ± 3 infective cercariae of* Sj* for 5, 6, 8, and 12 weeks were detected for TGF-*β*1 mRNA expression in* Sj* by using PCR (a) and SYBR Green-based quantitative PCR (b).* Sj* tubulin-*α* was detected as an input control. ^*∗*^ *P* < 0.05, ^*∗∗*^ *P* < 0.01. (c) Equal amounts of total RNA from infected mice livers at indicated time points were detected for mouse TGF-*β*1 mRNA expression by SYBR Green-based quantitative PCR. GAPDH was detected as an input control. ^*∗*^ *P* < 0.05, ^*∗∗*^ *P* < 0.01.

To gain a better understanding of the source of TGF-*β*1 in hepatic fibrogenesis, the transcriptional levels of TGF-*β*1 in mice and* Sj* were evaluated using RT-PCR and SYBR Green quantitative PCR.* Sj*-infected mouse liver cDNA was prepared as a PCR template, and the specific primer pairs shown in Figure 4(a) were also used. TGF-*β*1 mRNA expression level in* Sj* increased, especially at week 8 (Figures 5(a) and 5(b)), and this level was comparable with the TGF-*β*1 protein level in blood plasma and livers of infected mice, as shown in Figures 1(a) and 1(b). However, the mRNA expression level of mouse TGF-*β*1 decreased significantly in all time courses after* Sj* infection (Figure 5(c)). These results suggested that high protein level of TGF-*β*1 was transcribed mainly from* Sj*, but not from mice. This finding was consistent with the low transcriptional level of TGF-*β*1 in the liver during* Sj* infection, as reported by Bartley et al. [35], although Bartley et al. did not test TGF-*β*1 from the parasite.

## 4. Discussion

Infection with the parasitic helminth* Schistosoma* accounts for a significant portion of liver fibrosis cases in humans. The causative factors of hepatic fibrogenesis and the host-parasite interaction mechanisms need to be elucidated. TGF-*β*1 is one of the strongest factors promoting liver fibrosis by activating HSC [2–4].* Sj*-infected mouse model did not exhibit high TGF-*β*1 transcription level, but chronic schistosomiasis patients showed high TGF-*β*1 transcription level compared with healthy individuals [35, 36]. Hirata et al. [24] revealed the expression of TGF-*β*1-, TGF-*β*2-, and TGF-*β*3-like molecules in* Sj*. Some members of TGF-*β* superfamily, including InACT, BMP, receptors of TGF-*β*, and Smad1, Smad2, and Smad4 and other signaling pathway-associated molecules have been identified in* Schistosoma* [14–22]. In addition, tTG is the principal enzyme controlling TGF-*β* maturation rate [29–31]. Upregulation of tTG increases the concentrations of active TGF-*β* in various pathological states [31–33]. Thus, we systemically detected the level and source of TGF-*β*1, its relationship with tTG, and the extent of liver fibrosis in mice.

Our results showed that a high level of TGF-*β*1 mature peptide existed in liver tissue and blood stream, and the overexpression of TGF-*β*1 in* Sj*-infected liver section was mainly observed in cells near the* Sj* adult worms parasites or the deposited* Sj* eggs. TGF-*β*1 was downregulated by tTG inhibitor CTM treatment. The protein level of TGF-*β*1 mature peptide was highly consistent with the level of tTG protein and activity. In mouse models and human patients with alcoholic steatohepatitis, tTG provokes hepatocyte death and is associated with alcohol-induced liver fibrosis [37–39]. Our previous study demonstrated that HSC of* Sj*-infected-mice liver were activated and the extent of liver fibrosis gradually worsened from week 5 to week 12, and these changes were associated with tTG and IL-13 levels [34]. This study indicated that tTG-regulated TGF-*β*1 is also correlated with liver fibrosis.

IHC, qPCR, and ELISA results revealed the presence of TGF-*β*1 in* Sj* adult worm sections,* Sj* eggs, and the cultured medium of* Sj* adult worms. Furthermore, we extended and cloned the sequence of TGF-*β*1 in* Sj*. Although* Sj* whole-genome shotgun (WGS) sequence has been loaded into the NCBI GenBank [40], we failed to find any identical or even similar sequence using NCBI BLASTN to analyze the homology of the extended TGF-*β*1 cDNA sequence with the* Sj* WGS sequence. We also could not amplify its DNA sequence using* Sj* DNA as template and many alternative primer pairs. Parasites were nutritionally dependent on their host organisms and generally have an intimate, long-term physical association with their hosts [41]. Horizontal gene transfer (HGT) is rampant in prokaryotes [42]. Numerous independent studies have implicated schistosomes as agents (donors or recipients) of HGT [43]. Schistosomes cover their body surface with host antigens to avoid being detected by the host's immune system [44, 45] and this form of molecular mimicry might be due to HGTs [46–50]. Retroviruses such as transposable elements (TEs) may have the capacity to transfer genes [51]. Long terminal repeat (LTR) retrotransposons encode envelope-like proteins that provide infective capacity similar to viruses [52, 53]. Schistosome genomes are relatively large and known to be rich in TEs. Approximately half of their genetic material consists of TEs and repeat sequences, including LTR retrotransposons [26, 40, 54]. Therefore, TGF-*β*1 in* Sj* might be transferred through HGTs or retroviruses (TEs). Multiple lines of evidence are needed for conclusive documentation while avoiding false positives.

High transcription level of* Sj*-specific TGF-*β*1, but not mice-specific TGF-*β*1, was consistent with the extent of liver fibrosis. tTG, being the principal enzyme, controls the rate of TGF-*β*1 maturation [29–31]. Our previous report also confirmed that tTG is involved in the development of* Sj* infection-induced liver fibrosis in mice, and the mechanism may be associated with tTG-regulated IL-13 expression [34]. Thus, we demonstrated the relationship between tTG and TGF-*β*1. In our study, TGF-*β*1 is upregulated in* Sj-*infected mice liver (Figure 1), and TGF-*β*1 level is suppressed by CTM along with* Sj*-induced liver fibrosis remission (Figure 2), thereby suggesting that tTG-regulated* Sj* TGF-*β*1 is involved in liver granuloma and fibrosis in* Sj-*infected mice.

In summary, we confirmed that tTG-regulated TGF-*β*1 and IL-13 were associated with liver fibrosis in mice after* Sj*-infection; thus, tTG might serve as a potent treatment target. Whether TGF-*β*1 was of* Sj* and not of host origin requires further study.

## 5. Conclusion

In* Sj*-infected mice, TGF-*β*1 level in* Sj* is regulated by tTG and is consistent with the extent of liver granuloma and fibrosis. The origin or transfer route of TGF-*β*1 gene in* Sj* needs to be confirmed in the future. Host tTG helps with TGF-*β*1 maturation in the parasite, thereby showing that tTG is probably a potential drug target.
